# NPM1 Gene Type A Mutation in Bulgarian Adults with Acute Myeloid Leukemia: A Single-Institution Study

**DOI:** 10.4274/Tjh.2013.0023

**Published:** 2014-03-05

**Authors:** Gueorgui Balatzenko, Branimir Spassov, Nikolay Stoyanov, Penka Ganeva, Tihomit Dikov, Spiro Konstantinov, Vasil Hrischev, Malina Romanova, Stavri Toshkov, Margarita Guenova

**Affiliations:** 1 National Specialized Hospital for Active Treatment of Hematological Diseases, Laboratory of Cytogenetics and Molecular Biology, Sofia, Bulgaria; 2 Center of Excellence for Translational Research in Hematology, Sofia, Bulgaria; 3 National Specialized Hospital for Active Treatment of Hematological Diseases, Hematology Clinic, Sofia, Bulgaria; 4 National Specialized Hospital for Active Treatment of Hematological Diseases, Laboratory of Hematopathology and Immunology, Sofia, Bulgaria; 5 Medical University of Sofia, Faculty of Pharmacy, Department of Pharmacology, Toxicology and Pharmacotherapy, Sofia, Bulgaria

**Keywords:** Acute myeloid leukemia, NPM1 gene type A mutation, FLT3-ITD, allele-specific polymerase chain reaction

## Abstract

**Objective:** Mutations of the nucleophosmin (NPM1) gene are considered as the most frequent acute myeloid leukemia (AML)-associated genetic lesion, reported with various incidences in different studies, and type A (NPM1-A) is the most frequent type. However, since most series in the literature report on the features of all patients regardless of the type of mutation, NPM1-A(+) cases have not been well characterized yet. Therefore, we evaluated the prevalence of NPM1-A in Bulgarian AML patients and searched for an association with clinical and laboratory features.

**Materials and Methods:** One hundred and four adults (51 men, 53 women) were included in the study. NPM1-A status was determined using allele-specific reverse-transcription polymerase chain reaction with co-amplification of NPM1-A and β-actin and real-time quantitative TaqMan-based polymerase chain reaction. Patients received conventional induction chemotherapy and were followed for 13.2±16.4 months.

**Results:** NPM1-A was detected in 26 (24.8%) patients. NPM1-A mutation was detected in all AML categories, including in one patient with RUNX1-RUNX1T1. There were no differences associated with the NPM1-A status with respect to age, sex, hemoglobin, platelet counts, percentage of bone marrow blasts, splenomegaly, complete remission rates, and overall survival. NPM1-A(+) patients, compared to NPM1-A(-) patients, were characterized by higher leukocyte counts [(75.4±81.9)x109/L vs. (42.5±65.9)x109/L; p=0.049], higher frequency of normal karyotype [14/18 (77.8%) vs. 26/62 (41.9%); p=0.014], higher frequency of FLT3-ITD [11/26 (42.3%) vs. 8/77 (10.4%); p=0.001], and lower incidence of CD34(+) [6/21 (28.8%) vs. 28/45 (62.2%); p=0.017]. Within the FLT3-ITD(-) group, the median overall survival of NPM1-A(-) patients was 14 months, while NPM1-A(+) patients did not reach the median (p=0.10).

**Conclusion:** The prevalence of NPM1-A mutation in adult Bulgarian AML patients was similar to that reported in other studies. NPM1-A(+) patients were characterized by higher leukocyte counts, higher frequency of normal karyotypes and FLT3-ITD, and lower incidence of CD34(+), supporting the idea that the specific features of type A mutations might contribute to the general clinical and laboratory profile of NPM1(+) AML patients.

## Abstract (tr)

**Amaç:** Nukleofosmin (NPM1) genine ait mutasyonlar değişik çalışmalarda farklı insidanslar bildirilmekle birlikte, akut miyeloid lösemi (AML)-ilişkili genetik bozuklukların en sık görülenidir ve bunlar arasında tip A (NPM1-A) en sık rastlanan tipidir. Ancak literatürdeki serilerin çoğu mutasyon tipinden bağımsız olarak tüm hastaların özelliklerini sunmakta olup, NPM1-A(+) olgular bundan önce ayrıntılı olarak tanımlanmamıştır. Bu yüzden, Bulgar AML hastalarında NPM1-A prevalansı değerlendirilmiş, klinik ve laboratuvar özellikler ile ilişkileri araştırılmıştır.

**Gereç ve Yöntemler:** Çalışmaya yüz dört hasta (51 erkek, 53 kadın) dahil edilmiştir. NPM1-A durumu allel-özgül polimeraz zincir reaksiyonu ile NPM1-A ve b-aktinin birlikte amplifikasyonu ve gerçek-zamanlı TaqMan-bazlı polimeraz zincir reaksiyonu ile belirlenmiştir. Hastalar konvansiyonel indüksiyon kemoterapisi almış ve 13,2±16,4 ay takip edilmişlerdir.

**Bulgular:** NPM1-A 26 hastada (%24,8) tespit edildi. NPM1-A mutasyonu, RUNX1-RUNX1T1 taşıyan bir hastayı da içeren tüm AML kategorilerinde gösterilmiştir. Yaş, cinsiyet, hemoglobin, trombosit sayısı, kemik iliğindeki blast yüzdesi, splenomegali, tam remisyon oranları ve genel sağkalım ile NPM1-A durumu ile ilişkili farklılık saptanmadı. NPM1-A(+) hastalar, NPM1-A(-) hastalar ile karşılaştırıldıklarında daha yüksek lökosit sayısına [(75,4±81,9)x10^9^/L vs. (42,5±65,9)x10^9^/L; p=0,049], daha sık normal karyotipe [14/18 (%77,8) vs. 26/62 (%41,9); p=0,014], daha sık FLT3-ITD’ye [11/26 (%42,3) vs. 8/77 (%10,4); p=0,001] sahipti ve daha seyrek CD34 (+) [6/21 (%28,8) vs. 28/45 (%62,2); p=0,017] idi. FLT3-ITD(-) grup içinde, NPM1-A(-) hastaların ortanca genel sağkalımı 14 aydı, ancak NPM1-A(+) hastalar ortancaya ulaşmadı (p=0,10).

**Sonuç:** Erişkin Bulgar hastalarda NPM1-A mutasyonunun prevalansı diğer çalışmalarda bildirilenlerle benzer bulundu. NPM1-A(+) hastalar, tip A mutasyonların spesifik özelliklerinin NPM1(+) AML hastaların genel klinik ve laboratuvar profiline katkıda bulunabileceği fikrini destekler nitelikte yüksek lökosit sayısı, daha sık normal karyotip ve FLT3-ITD ile daha seyrek CD34(+) sıklığı ile karakterizeydi.

## INTRODUCTION

Acute myeloid leukemia (AML) is a heterogeneous group of clonal disorders with great variability in terms of pathogenesis; morphological, genetic, and immunophenotypic characteristics of the leukemic blast population; clinical course; and response to therapy. It is believed that this heterogeneity is largely related to variations in the spectrum of the underlying molecular abnormalities that alter normal cellular mechanisms of self-renewal, proliferation, and differentiation [1]. 

Several lines of evidence support the idea that hematopoietic precursor cells in AML undergo malignant transformation in a multistep process of acquisition of different genetic abnormalities that might range from relatively large chromosome alterations to single nucleotide changes, deregulated gene expression, or epigenetic changes [2]. Some of these abnormalities exhibit strong correlations with the phenotypic features of the disease and/or treatment outcome and define biologically and prognostically different subtypes of AML, as recognized in the latest World Health Organization (WHO) classification system in 2008. The category of “AML with recurrent genetic abnormalities” consists of 6 subtypes, characterized by specific chromosome translocations that lead to the formation of fusion genes. Additionally, 2 provisional entities, AML with mutated nucleophosmin (NPM1) and AML with mutated CCAAT/enhancer binding protein alpha (CEBPA), have also been recognized [3]. The former is considered as the most frequent AML-associated genetic lesion.

The NPM1 gene maps to chromosome 5q35 and encodes a ubiquitously expressed chaperone protein that shuttles between the nucleus and cytoplasm but predominantly resides in the nucleus. It is involved in multiple functions and plays key roles in ribosome biogenesis, centrosome duplication, genomic stability, cell cycle progression, and apoptosis [4].

NPM1 is frequently overexpressed in solid tumors [5], while in hematological malignancies, the NPM1 locus is lost [6] or translocated, leading to the formation of fusion genes and proteins [7]. Recently somatic mutations in exon 12 of the NPM1 gene have been found in approximately one-third of all adult patients with AML [4,8]. Mutations of the NPM1 gene induce delocalization of the NPM1 protein in AML, while in solid tumors, only NPM1 overexpression, but not delocalization, has been reported so far [9]. Some differences in the incidence of NPM1 mutations were observed, suggesting the possible influence of ethnic and geographic factors [10,11]. Therefore, data concerning the incidence of the molecular abnormality in particular countries might be helpful in the analysis of the impact of local factors.

As reported so far, NPM1-mutation-positive patients are more often females, with a normal karyotype, and usually present with high white blood cell (WBC) counts and higher percentages of bone marrow blasts, frequently with myelomonocytic or monocytic morphology, with absent or low expression of CD34, and with frequent FLT3 mutations [12,13]. The presence of NPM1 mutations is associated with unique gene expression [14] and microRNA profiles [15]. NPM1 mutations predict an excellent response to induction therapy [12] and provide important prognostic information as stable markers for minimal residual disease monitoring in AML patients [16,17].

Currently there are 55 described mutations of NPM1 exon 12 in AML that result in similar alterations at the C-terminus of the mutant proteins. The most prevalent types of mutations are mutation A (75%-80%), mutation B (10%), and mutation D (5%), while all other mutations are very rare [4,18]. To date, most studies have focused on the clinical and laboratory profile of all NPM1-mutated AML patients regardless of the type of the mutation, and, therefore, the clinical and laboratory characteristics of patients, particularly those with the most frequent type A mutations, have not been precisely recorded. Certain differences might be expected since some studies suggested that the outcome and prognosis in patients with type A and non-A mutations might not be identical [19,20]. Therefore, in this study we performed molecular screening aiming at establishing the prevalence of type A mutation of the NPM1 gene in Bulgarian adult AML patients and searched for an association with major clinical and laboratory features commonly reported in literature. 

## MATERIALS AND METHODS

**Patients**

The NPM1 type A [NPM1-A] mutation was studied in the bone marrow cells of 104 adults (51 men, 53 women) at a mean age of 53.7±15.8 years (range: 22-82 years), diagnosed and treated at the National Specialized Hospital for Active Treatment of Hematological Diseases, Sofia, Bulgaria, after receiving informed consent.

The diagnosis of AML was based on WHO 2008 classification criteria using a combination of clinical data and morphological, cytochemical, flow cytometric, and/or immunohistochemical, cytogenetic, and molecular features. 

**Analysis of NPM1-A Mutation by Reverse-Transcription Polymerase Chain Reaction (RT-PCR)**

At the time of diagnosis, bone marrow mononuclear cells were separated after red blood cell destruction with a lysis buffer (155 mM NH4Cl, 10 mM KHCO3, 0.1 mM EDTA). Total cellular RNA was isolated using TRIzol Reagent (Invitrogen, Karlsruhe, Germany) according to the manufacturer’s protocol. cDNA was synthesized by reverse transcription of 1 µg of RNA in a reaction medium with a final volume of 20 µL containing 1X first-strand buffer, 200 U of MMLV reverse transcriptase (USB Products, Affimetrics, Cleveland, OH, USA), 1 mM of each deoxynucleoside-5’-triphosphate (dNTP), 20 U of RNase Inhibitor (Invitrogen), and 5 µM of random hexamers (Thermo Scientific, Waltham, MA, USA), by consecutive incubation of the samples at 37 °C for 1 h and at 99 °C for 3 min. 

The presence of NPM1-A mutation was determined using 2 different PCR approaches. In the first, allele-specific PCR was carried out by simultaneous amplification of NPM1-A with allele-specific primers and β-actin cDNA as an internal control. Briefly, 3 µL of cDNA was amplified in a reaction medium with a final volume of 25 µL containing 1X PCR buffer, 2.5 mM MgCl2, 200 µM of each dNTP, 1 U of Taq polymerase (Promega, Madison, WI, USA), and 10 pmol of each of the following primers: NPM1-mutA (F): 5′-caagaggctattcaagatctctgtctg-3’ and NPM-REV-6 (R): 5’-accatttccatgtctgagcacc-3’ (NPM1-A), together with b-actin (S) 5’-ggcatcgtgatggactccg-3’ and b-actin (AS) 5’-gctggaaggtggacagcga-3’ (β-actin). The reaction started with denaturation at 95 °C for 7 min; proceeded with 35 cycles of amplification at 95 °C for 45 s, at 67 °C for 45 s, and at 72 °C for 45 s; and terminated at 72 °C for 7 min on a Veriti Thermal Cycler (Applied Biosystems, Foster City, CA, USA). Amplification products were run in 2% (w/v) agarose gel, stained with ethidium bromide, and visualized after UV exposure. The second approach employed real-time quantitative TaqMan-based PCR using the MutaQuant® Kit NPM1 mutation A (Ipsogen, Marseille, France) following the manufacturer’s instructions on a Rotor-Gene 6000 thermocycler (Corbett Life Science, Mortlake, Australia).

**Treatment**

Sixty-three patients with non-acute promyelocytic leukemia (non-APL) received conventional induction chemotherapy with one of the anthracyclines (doxorubicin or idarubicin) for 3 days and cytosine arabinoside for 7 days. Patients with APL received all-trans retinoic acid with or without concurrent induction chemotherapy. After complete remission was achieved, patients received consolidation chemotherapy with conventional doses of cytosine arabinoside and one anthracycline or with high-dose cytosine arabinoside. The mean period of follow-up of treated patients was 13.2±16.4 months.

Five of the patients died before the start of any treatment. Early death during the first induction course occurred in 16 (15.4%) patients. Due to old age and/or poor performance status, no chemotherapy or only low-dose cytosine arabinoside was given in 16 patients. One patient was lost from contact. 

**Statistical Analysis**

All statistical analysis was performed using SPSS 16.0.1. The Wilcoxon Mann-Whitney test was used to compare the distributions of numerically valued variables. Univariate differences between categorical variable subsets were evaluated with Fisher’s exact test. Overall survival (OS) was estimated for patients who received at least one induction course of therapy using the Kaplan-Meier method. Two-sided p<0.05 was considered to be of statistical significance. 

## RESULTS

**Figure 1:**
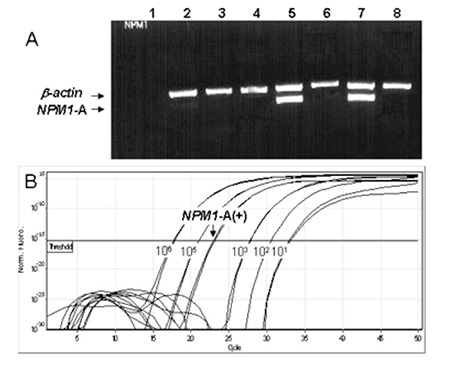
Pattern of detection of NPM1-A mutation by reverse-transcription polymerase chain reaction (RT-PCR). A) Detection of NPM1-A mutation by allele-specific RT-PCR:1=negative control; 5,7=NPM1-A(+) patients; 2,3,4,6,8=NPM1-A(-) patients. B) Detection of NPM1-A mutation by quantitative real-time RT-PCR.

A positive reaction for NPM1-A mutation [NPM1-A(+)] by both approaches was detected in 26 of 104 (24.8%) patients (Figure 1). No discrepancies in the results generated by the 2 methods were observed.

There were no significant differences between NPM1-A(+) and NPM1-A(-) patients with respect to age, sex, hemoglobin, platelet counts, percentage of bone marrow blasts, or the presence of splenomegaly (Table 1). However, the mean WBC count was significantly higher in NPM1-A(+) compared to NPM1-A(-) patients at (75.4±81.9)x10^9^/L versus (42.5±65.9)x10^9^/L, respectively (p=0.049).

The statistical analysis did not show any significant differences in the frequency of the molecular abnormality in the defined AML categories. However, the incidence of NPM1-A(+) was clearly lower in AML with recurrent genetic abnormalities, at 1/11 (9.1%), compared to AML with myelodysplasia-related changes (AML-MRC) at 4/11 (36.4%), therapy-related myeloid neoplasms at 3/15 (20.0%), and AML not otherwise specified (NOS) at 15/65 (27.7%). 

The tendency for a lower frequency of NPM1-A mutations in patients with recurrent genetic abnormalities was even more prominent when all patients regardless of previous chemo- and/or radiotherapy were analyzed. Thus, a total of 17 patients comprising 11 de novo cases and 6 therapy-related AML cases with fusion transcripts (PML-RARA, n=2; RUNX1-RUNX1T1, n=3; CBFb-MYH11, n=1) included only 1 (5.9%) positive case, versus 25/87 (28.7%) in the remaining group of patients (p=0.064).

Among the different subtypes of AML and NOS cases, no statistical differences in the prevalence of NPM1-A(+) were observed, with a relatively higher value in AML without maturation [6/13 (46.2%)] and in AML with maturation and acute myelomonocytic leukemia [4/11 (36.4%) and 5/16 (31.2%), respectively]. No positive reaction for NPM1-A was found in patients with AML with minimal differentiation, acute erythroid leukemia, and acute megakaryoblastic leukemia; however, the number of studied cases was too low for more general conclusions.

Immunophenotyping of patients with and without NPM1-A revealed statistically lower frequency of CD34(+) in NPM1-A(+) compared to NPM1-A(-) patients, at 6/21 (28.8%) versus 28/45 (62.2%), respectively (p=0.017), while no differences were observed in regard to aberrant co-expression of lymphoid antigens or CD56.

The overall incidence of NPM1-A mutation among patients with a normal karyotype was 14/40 (35%). Interestingly, within the NPM1-A(+) group, a distinct overrepresentation of patients with a normal karyotype [14/18 (77.8%) vs. 26/62 (41.9%); p=0.014] and internal tandem duplication of the FLT3 gene (FLT3-ITD) [11/26 (42.3%) vs. 8/77 (10.4%); p=0.001] was observed compared to patients without the mutation. No association between NPM1-A status and the presence of partial tandem duplication of the MLL gene (MLL-PTD) and overexpression of the EVI1 gene was detected. 

Sixteen patients (15.4%) died within the first month after diagnosis; however, no differences in the early death rates between the NPM1-A(+) and NPM1-A(-) groups were observed [5/24 (20.8%) and 11/75 (14.7%), respectively; p=0.16]. 

Overall, a complete remission was achieved in 38 of 66 (57.6%) patients, including 11 out of 16 (68.7%) NPM1-A(+) patients and 27 out of 50 (54.0%) NPM1-A(-) patients (p=0.24).

**Figure 2:**
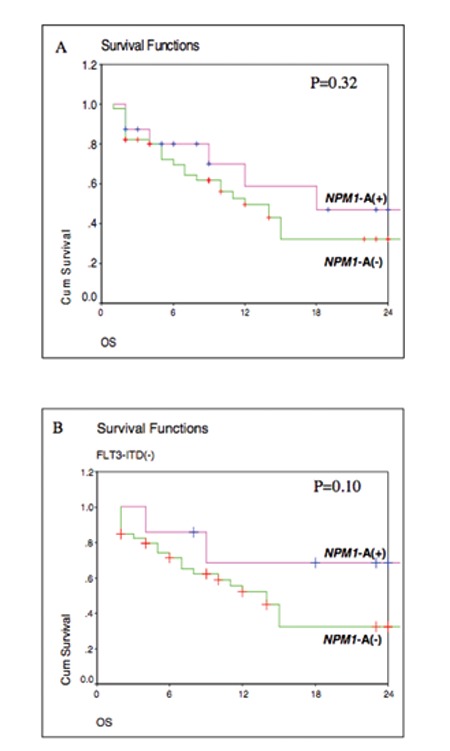
Kaplan-Meier survival curves. A) OS of NPM1-A(+) and NPM1-A(-) AML patients. B) OS of NPM1-A(+) and NPM1-A(-) AML patients within the group of patients without FLT3-ITD.

The median OS for NPM1-A(+) non-APL patients was 18.0 months, compared to 12.0 months for NPM1-A(-) non-APL patients [log rank test, p=0.322]. When patients were additionally stratified according to their FLT3-ITD status, we found that, within the FLT3-ITD(-) group, the median OS of patients without the NPM1-A mutation was 14 months, while NPM1-A(+) patients did not reach this median. Due to the relatively small number of patients, this tendency was still not statistically significant (p=0.10). Within the FLT3-ITD(+) group, the median OS for NPM1-A(+) and NPM1-A(-) was 12 months and 5 months, respectively (p=0.88) (Figure 2).

## DISCUSSION

In this study, we screened 104 adult Bulgarian patients with AML for NPM1 gene type A mutation using 2 different RT-PCR based approaches and positive results by both methods were found in 24.8% of patients. This result was similar to previously reported frequencies of 19.1%-20.3% for NPM1-A [21,22], while the incidence of all forms of NPM1 mutations in adults varied from 24.9% to 34.5% in the literature [22]. 

Several studies, including ours, clearly demonstrated that the frequency of NPM1 gene mutations is significantly higher in AML patients with a normal karyotype [22], despite results being heterogeneous and varying from 38.1% to 63.8% [22,23]. The data concerning the incidence of NPM1-A mutation in particular within the category of patients with normal karyotypes are still scarce, mainly because the reported data encompass the whole spectrum of NPM1 gene mutations in most of the studies and only in a few of them did the authors specify the frequency of type A mutation. In our study, we found NPM1-A mutations in 35% of normal-karyotype patients, similar to the results reported by Schnittger et al. (41.4%) [24] and Döhner et al. (36.7%) [13]. 

The group of NPM1-A(+) patients in our study was characterized by a higher WBC count at diagnosis (p=0.049), higher frequency of normal karyotypes (p=0.012) and FLT3-ITD (p=0.001), and lower incidence of CD34(+) (p=0.017). These results corresponded to the characteristics of patients with NPM1 gene mutations generally described in the literature regardless of the type of mutation [25,26]. 

In contrast, several other findings in our study differed from those of other reports. According to some authors, NPM1 mutations occur almost exclusively in de novo AML cases [25], while, in our study, the abnormality was observed in 20% of therapy-related AML cases, confirming recently published data that 16% of patients with therapy-related AML are also positive for NPM1 mutations [27]. Presumably the presence of NPM1 mutations in some cases might be associated with the development of de novo AML, regardless of the impact of the prior radio-/chemotherapy [28]. 

Earlier, it was suggested that NPM1 mutations and recurrent genetic abnormalities are mutually exclusive in AML patients [29]. However, our study demonstrated at least one patient with simultaneous co-expression of NPM1-A and RUNX1-RUNX1T1 transcripts. Occasionally, similar cases were reported by others, both in adults and children [18,21,23,30]. Errors in sample registration, PCR contamination, or other technical factors might explain these findings in some [29], but not all, of these cases. Therefore, several questions, such as whether NPM1 mutations and concurrent genetic abnormalities occur in the same or different leukemic cell populations and whether the occurrence of 2 or more specific genetic markers in exceptional cases is just coincidental or represents a true association, are still not understood [29].

Other variables that are still a subject of controversy are the sex- and age-associated differences in the incidence of NPM1 mutation. Previously, a significantly higher incidence of NPM1 mutations in females was reported by Thiede et al. [18] and Falini et al. [7]; however, these observation were not confirmed by our study or others [21,31]. Similarly, according to Schneider et al. [32], NPM1 mutations significantly decreased with age, while others reported that patients with NPM1 mutations were older than those without the mutation [11,21,33]. In our study, as well as in those of Döhner et al. [13] and Luo et al. [31], no age-associated differences in the NPM1-A mutation status were found. Several factors might contribute to the heterogeneity of the obtained results, such as variations in the biological characteristics of patients (whole AML group vs. AML patients with normal cytogenetics) or in the applied method for NPM1 gene mutation detection and the methodological technical variables [29].

In our study, in addition to AML-MRC and therapy-related AML, NPM1-A has been also detected in AML without maturation, AML with maturation, acute myelomonocytic leukemia, and acute monoblastic/monocytic leukemia within the category of AML-NOS without significant differences in the incidence among the various subtypes (p=0.50). Previously, it has been suggested that NPM1 mutations could be found in different AML French-American-British (FAB) entities [23], with a higher frequency in the M4/M5 subtypes [13,24]. Mutations were never found in FAB M3 and were less common in M0, M4eo, M6, and M7 [18], in agreement with our data. However, according to Luo et al., in AML patients with normal cytogenetics, there was no correlation between NPM1 mutations and FAB morphologic subtypes, with a positive reaction for NPM1 predominantly in M2 and M5 cases [31]. Interestingly, in a study of 252 NPM1-positive patients, those with AML M5 represented only 12.7% of the whole group, while the majority of patients had AML M1 (21.9%), AML M2 (25.1%), and AML M4 (27.9%) morphology [34].

Within the category of AML-MRC, we found NPM1-A(+) in 36.4% of patients, similarly to Döhner et al., who found 5 patients with NPM1 mutations out of 13 (38.5%) with secondary AML following myelodysplastic syndrome [13]. In contrast, Devillier et al. reported positive results in only 8% of AML-MRC cases [35], while Falini et al. initially reported that NPM1 gene mutations were found only in de novo AML and not in the 135 AML cases arising from myelodysplasia [12]. It is difficult to explain the reasons for these differences. First, the number of analyzed cases in our cohort of patients, as well as in that reported by Döhner et al. [13], was too low for definitive conclusions concerning the real incidence. On the other hand, the category of AML-MRC consists of 3 subtypes, including cases with previous history of Myelodysplastic syndrome, cases with Myelodysplastic syndrome-related cytogenetic abnormality, and cases with multilineage dysplasia [36]. Depending on the prevalence of the particular subtype, the incidence of NPM1 mutations may vary. Regardless of the precise frequency, the identification of these patients is important from a clinical point of view in 2 aspects: first, in regard to the classification as AML-MRC (applying the WHO morphologic criteria) or as AML with NPM1 mutation (using the WHO genetic criteria), and second, because multilineage dysplasia has no impact on the biologic, clinicopathologic, and prognostic features of AML with mutated nucleophosmin [37].

In a number of studies, a favorable impact of NPM1 gene mutations, particularly of type A mutations, on the outcome was reported [21]. In this study we did not find significant differences between NPM1-A(+) and NPM1-A(-) patients with regard to achievement of complete remission (CR) and OS, despite a clear tendency for better treatment response being observed in the group of patients with concomitant FLT3-ITD. Similarly, no differences in CR rates between NPM-mutated and NPM wild-type patients were reported by Boissel et al. [38]. Several factors might have an impact on these results, such as the overall efficiency of the applied treatment protocols, the rate of intensive induction course approaches [38], the patients’ ages [39], or the presence of other molecular abnormalities.

In conclusion, the prevalence of NPM1-A mutations in adult Bulgarian AML patients was similar to that reported by other studies. NPM1-A(+) patients in our study were characterized by higher leukocyte counts at diagnosis, higher frequency of normal karyotypes, higher frequency of FLT3-ITD, and lower incidence of CD34(+) immunophenotypes, supporting the idea that the specific features of type A mutations of the gene might contribute to the general clinical and laboratory profiles of AML patients with NPM1 mutations.

## ACKNOWLEDGMENTS

This study was supported by a grant from the National Research Fund, Bulgarian Ministry of Education and Science (D02-35/2009).

## CONFLICT OF INTEREST STATEMENT

The authors of this paper have no conflicts of interest, including specific financial interests, relationships, and/ or affiliations relevant to the subject matter or materials included.

## Figures and Tables

**Table 1:**
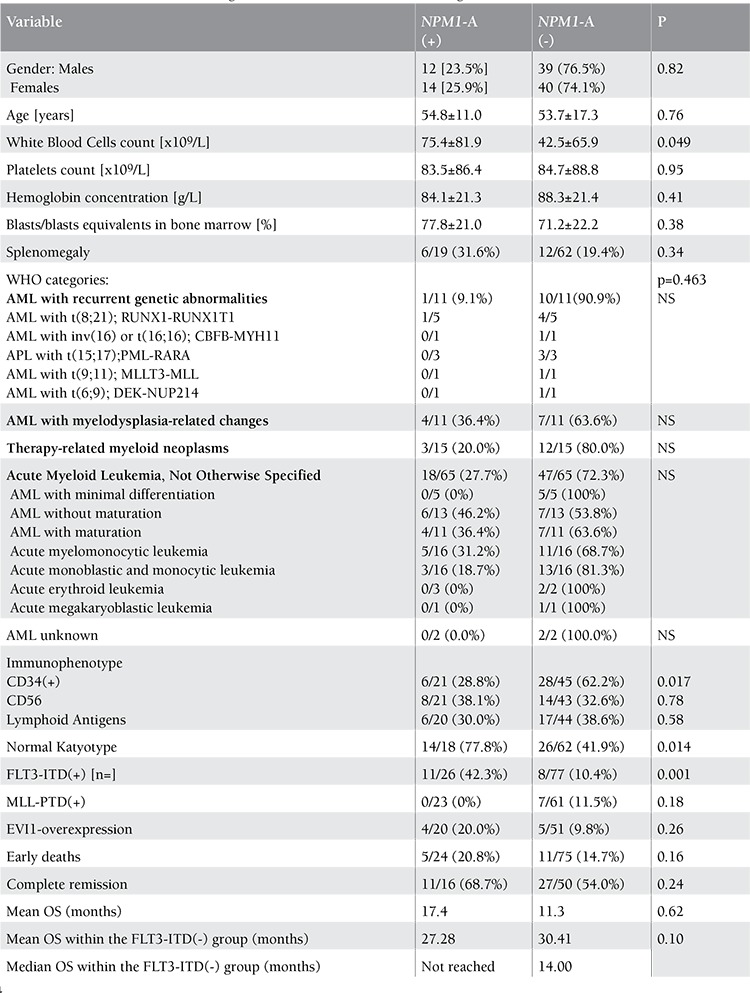
Patient characteristics according to NPM1-A mutation status. NS: Not significant.
